# Supplementary material for: Development of Water-Compatible Molecularly Imprinted Polymers Based on Functionalized β-Cyclodextrin for Controlled Release of Atropine
Source: Polymers (Basel). 2020 Jan 6;12(1):130. doi: 10.3390/polym12010130 (PMC7022701; doi:10.3390/polym12010130)
Supplement: Supplementary file 1 [file polymers-12-00130-s001.pdf]

Electronic Supporting Material

Development of water-compatible molecularly imprinted polymers based on functionalized β-cyclodextrin for controlled release of atropine

Yahui He[a,b*], Shaomei Zeng[b], A. M. Abd El-Aty[c,d,e,*], Ahmet Hacımüftüoğlu[e], Woldemariam Kalekristos Yohannes[a] , Majid Khan[a], Yongxin She[b*]

[a]*Beijing Technology and Business University, Beijing 100048, P.R. China*

[b]*Institute of Quality Standards & Testing Technology for Agro-Products, Chinese Academy of Agricultural Sciences, Beijing 100081, P.R. China*

[c]*State Key Laboratory of Biobased Material and Green Papermaking, College of Food Science and Engineering, Qilu University of Technology, Shandong Academy of Science, Jinan 250353, China*

[d]*Department of Pharmacology, Faculty of Veterinary Medicine, Cairo University, 12211-Giza, Egypt*

[e]*Department of Medical Pharmacology, Medical Faculty, Ataturk University, 25240-Erzurum, Turkey*

***Correspondence**: hyh@btbu.edu.cn (Y.H.); abdelaty44@hotmail.com (A. M. Abd El-Aty), sheyongxin@caas.cn (Y.S.).

Table S1 Comparison of the performance of the developed MIP in the present study and some releasing MIP reported in literature.

**MIP for targets**	**Functional Monomer**	**Qm ($mg \cdot g^{-1}$)**	**Drug release rate (%/24h)**	**Release environment**	**Ref.**
Vancomycin	DEAEMA	17	80	5.2 and 7.4	Mao, et al. (2017)
Doxorubicin hydrochloride	MAA	166.28	50	7.4	(Chen, et al. (2016)
Azithromycin	MAA	127	24	7.4	(Sheybani, et al. (2015)
Dichlorophenol	MAA-β-CD	45.67	-	-	(Surikumaran, et al. (2014)
Sinomenine hydrochloride	MAA+β-CD	93.8	78	7.4	(Chen, .et al. (2018))
Atropine	MAA-β-CD	240	65	1.5 and 7.4	This work

References

Congyang Mao , Xianzhou Xie , Xiangmei Liu , Zhenduo Cui , Xianjin Yang , K.W.K. Yeung , Haobo Pan, Paul K. Chu , Shuilin Wu.The controlled drug release by pH-sensitive molecularly imprinted nanospheres for enhanced antibacterial activity. Materials Science and Engineering C 77 (2017) 84–91.

Fangfang Chen, Heng Chen, Xiao Duan, Jiqiong Jia, and Jie Kong.Molecularly imprinted polymers synthesized using reduction-cleavable hyperbranched polymers for doxorubicin hydrochloride with enhanced loading properties and controlled release.J Mater Sci (2016) 51:9367–9383。

Simin Sheybani, Tolou Hosseinifar, Majid Abdoussand Saeedeh Mazinani .Mesoporous molecularly imprinted polymer nanoparticles as a sustained release system of azithromycin.RSC Adv., 2015, 5, 98880–98891.

Hemavathy Surikumaran, Sharifah Mohamad * and Norazilawati Muhamad Sarih.Molecular Imprinted Polymer of Methacrylic Acid Functionalised β-Cyclodextrin for Selective Removal of 2,4-Dichlorophenol.Int. J. Mol. Sci. 2014, 15, 6111-6136.

Hanqiu Chen，Wen Zhang，Ning Yang，Chongmin Chen，Mingliang Zhang.Chitosan-Based Surface Molecularly Imprinted Polymer Microspheres for Sustained Release of Sinomenine Hydrochloride in Aqueous Media.Appl Biochem Biotechnol (2018) 185:370–384.
